# The relationship between childhood adversity and affective instability across psychiatric disorders: A meta‐analysis

**DOI:** 10.1111/acps.13745

**Published:** 2024-08-11

**Authors:** Jasper Palmier‐Claus, Rebecca Golby, Laura‐Jean Stokes, Christopher W. N. Saville, Kyriakos Velemis, Filippo Varese, Steven Marwaha, Elizabeth Tyler, Peter Taylor

**Affiliations:** ^1^ Spectrum Centre for Mental Health Research, Faculty of Health & Medicine Lancaster University Lancaster UK; ^2^ Lancashire & South Cumbria NHS Foundation Trust Preston Lancashire UK; ^3^ Psychology Department, Faculty of Science & Technology Lancaster University Lancaster UK; ^4^ School of Psychology and Sports Science Bangor University Bangor UK; ^5^ Greater Manchester Mental Health NHS Foundation Trust Manchester UK; ^6^ Division of Psychology & Mental Health, School of Health Sciences University of Manchester, Manchester Academic Health Sciences Centre Manchester UK; ^7^ Institute for Mental Health, School of Psychology University of Birmingham Birmingham UK; ^8^ Birmingham and Solihull Mental Health NHS Trust Birmingham UK

**Keywords:** adversity, affective instability, emotion regulation, rapid cycling, trauma

## Abstract

**Introduction:**

Affective instability represents an important, transdiagnostic biobehavioural dimension of mental ill health and clinical outcome. The causes of affective instability remain unclear. This systematic review and meta‐analysis evaluated the extent to which exposure to childhood adversity is associated with affective instability across psychiatric disorders, and which forms of adversity are most strongly associated with affective instability.

**Methods:**

The review followed a published protocol (PROSPERO: CRD42020168676). Searches in Medline, Embase and PsychInfo identified studies using quantitative measures of childhood adversity and affective instability, published between January 1980 and July 2023. Data were analysed using a random effects meta‐analysis separately for each outcome, namely affective lability, emotion dysregulation, and rapid cycling. The Mixed‐Methods Appraisal Tool was used to appraise the quality of the literature.

**Results:**

The search identified 36 studies involving 8431 participants. All reports focused on cross‐sectional associations. We did not identify any prospective longitudinal research. The analysis showed small, but statistically significant effects of childhood adversity on affective lability (*r* = 0.09, 95% CI 0.02, 0.17), emotion dysregulation (*r* = 0.25, 95% CI 0.19, 0.32), and rapid cycling (OR = 1.39; 95% CI 1.14, 1.70). When considering adversity subtypes, emotional abuse showed the strongest effect on affective lability (*r* = 0.16, 95% CI 0.07, 0.24) and emotion dysregulation (*r* = 0.32, 95% CI 0.19, 0.44). Quality assessment scores were generally low. Most studies failed to control for confounding factors or offer assurances around the representativeness of the samples.

**Conclusions:**

The findings suggest that childhood adversity, particularly emotional abuse, is associated emotional instability in adulthood, but further prospective longitudinal research is needed to confirm causality. The findings have implications for the prevention and treatment of affective instability across psychiatric disorders.

**Summations**

- Childhood adversity was significantly associated with all forms of affective instability.
- Of the adversity subtypes, childhood emotional abuse showed the strongest association with affective lability and emotion dysregulation.

**Limitations**

- There were a limited number of studies exploring the impact of childhood adversity subtypes on rapid cycling.
- The review failed to identify any prospective longitudinal research limiting inferences about causality.

## 1 INTRODUCTION

Affective instability has been defined as ‘rapid oscillations of intense affect, with a difficulty in regulating these oscillations or their behavioural consequences’.^1^ It encompasses a variety of affect‐related phenomena, including increased emotional reactivity, rapid cycling between emotions, and switching between emotional states.^2^ Within the psychological and psychiatric literature, it is sometimes referred to as mood instability, emotional dysregulation, affective lability, and mood swings. Affective instability is common in the general population, but is particularly prevalent in people with psychiatric disorders, where it is associated with adverse outcomes, including psychosis, service use, and poor functioning.^3^, ^4^ There is evidence suggesting an association between affective instability and suicidality,^5^ theorised to be the result of repeated activation of normally latent affect‐driven suicide schema.^6^ In patients diagnosed with bipolar disorder, affective lability is associated with a lower likelihood of and longer times to recovery.^7^ There is a phenomenological and biological overlap in how affective instability presents across populations, suggesting possible underlying transdiagnostic processes.^8^ Understanding the causes of affective instability across psychiatric disorders may be valuable to improve clinical outcomes.

One proposed risk factor for affective instability is childhood adversity, including sexual, physical, and emotional abuse.^9^ Childhood adversity can have a profound and lasting impact on people's lives,^10^, ^11^ potentially increasing affective reactivity to everyday stressors.^12^ There is evidence that it has a long‐term impact on brain structures responsible for emotional regulation and control.^13^, ^14^, ^15^ Meta‐analyses have suggested an association between childhood adversity and disorders characterised by affective instability, including bipolar disorder^16^ and borderline personality disorder.^17^ However, it is unclear what aspects of these disorders might explain these associations. To date, there has been no meta‐analytic investigation of the association between childhood adversity and affective instability across psychiatric disorders. There is a suggestion in the literature that emotional abuse may be particularly related to affective instability^16^ and analyses exploring the effect of specific adversity subtypes is warranted.

### 1.1 Aims of the study

This systematic review and meta‐analysis aimed to investigate whether exposure to childhood adversity is associated with different metrics of affective instability across psychiatric disorders. It also examined which specific forms of adversity had the strongest association with affective instability.

## 2 METHODS

This review was carried out in accordance with the Preferred Reporting Items for Systematic Reviews and Meta‐Analyses (PRISMA) guidelines, with a pre‐published protocol (PROSPERO: CRD42020168676).

### 2.1 Searches

Systematic searches in Medline, Embase, and PsychInfo identified peer reviewed literature published between January 1980 and July 2023. The authors used blocks of search terms pertaining to childhood adversity and affective instability (Supplementary Table 1), informed by previous reviews.^16^, ^17^ They also screened the reference lists, articles citing the included manuscripts, and relevant reviews.^1^, ^18^ Where not available, we contacted the lead or corresponding author for a copy of the manuscript. Two researchers (RG, KV) independently screened all titles and abstracts with 96% agreement. Reports felt to be potentially eligible by either rater were then screened at the full article level by both researchers with 85% agreement. Discrepancy between raters was resolved through team consensus.

### 2.2 Eligibility

The inclusion criteria were: (i) a sample with a formal psychiatric diagnosis according to Diagnostic and Statistical Manual (e.g., DSM‐III, DSM‐IIIR, DSM‐IV, DSM‐IV‐TR, and DSM‐5) or the International Classification of Diseases (e.g., ICD‐9, ICD‐10, and ICD‐11), including all psychotic, mood, anxiety, eating, and personality disorders; (ii) a quantitative measure of childhood adversity (age <18) including sexual abuse, physical abuse, emotional abuse, physical neglect, emotional neglect, bullying, or death of parent(s); (iii) a quantitative measure of mood instability in adulthood using the definition provided by Marwaha and colleagues,^1^ and allowing for varied nomenclature (e.g., emotional dysregulation, affective lability, and rapid cycling); (iv) publication after 1980 to coincide with current classifications of mental disorder; and (v) sufficient statistical information on the association between variables from which to generate an effect size. In the absence of this information or where clarity around eligibility was required, we contacted the lead and/or corresponding author. All articles had to be written in the English language and published in a peer‐reviewed journal. We excluded studies focusing on neurological or substance misuse disorders. In the case of multiple analyses conducted on the same sample, the available or largest dataset was selected.

### 2.3 Data extraction

Two researchers (RG, JPC) independently extracted data from eligible manuscripts. A data extraction template was created in Excel for recording statistical information alongside methodological features of research thought to influence the computed effect sizes. This included the type and assessment of adversity and affective instability, and the presence of covariates in the analysis. There was matching data for 89% of reports and discrepancy was resolved through consensus with the wider team.

### 2.4 Quality assessment

The quality of eligible studies were assessed using the quantitative non‐randomised studies subscale of the Mixed‐Methods Appraisal Tool 2018 version.^19^ This assesses the representativeness of the sample, the appropriateness of the assessments, the completeness of the data, whether appropriate confounders were controlled for in the analysis, and whether the exposure occurred as intended. For the purpose of this review, ‘appropriate confounders’ was defined as at least controlling for age, gender, and socioeconomic status, as these could plausibly affect the strength of the observed effects. Two independent researchers (L‐JS, CS) independently provided quality assessment ratings with moderate levels of agreement (57%; *rho* = 0.23). Discrepancy was resolved through discussion and consensus with the wider team.

### 2.5 Statistical analysis

The search identified three conceptually distinct, but overlapping, forms of affective instability. Emotional dysregulation emphasises the person's lack of capacity to regulate or control affect and associated responses, typically in response to internal or external events, whereas affective lability refers to the degree to which emotions fluctuate over time.^1^ Rapid cycling refers to diagnostically meaningful shifts between episodes of depression and mania,^20^ and is typically measured as a binary variable (present/absent). Given the methodological and theoretical differences in the outcomes, analyses were undertaken separately for each form of affective instability. Analyses for emotion dysregulation and affective lability were based on correlation coefficients. This included effects of the *r*‐family, but also other information that could be converted to *r*. For the analysis, we converted *r* to Fisher's *Z* for pooling, before results were converted back to *r*.^21^ Effects for rapid cycling were odds ratios based on the literature always treating this as a binary variable. Adjusted effects were used in the meta‐analyses, but the authors conducted a sensitivity analysis to explore the impact of this on the findings. Where studies included multiple independent samples, these were treated as independent effects. Where multiple affective instability metrics were considered in the same study, we included them in the separate analyses for the different outcomes.

Reports typically provided multiple effect sizes relating to different adversity subtypes arising from the same sample. Calculating a global, overall effect size (one effect per report) can be problematic and less meaningful when the strength of the effect varies considerably across variables, which was true for the adversity subtypes in the identified current literature. However, analysing these together would violate the assumption of independent effects in meta‐analysis. Consequently, we used a random correlated‐effects model with small sample size correction,^22^ which uses robust variance estimation that allows for non‐independent effects to be included together. This analysis included effects from all studies for any adversity subtype. However, to avoid redundancy, we did not include effects relating to a total adversity score (i.e., a summed or average score across adversity sub‐types), alongside the adversity subtypes, unless this was the only effect available for that study. A conservative estimated correlation of 0.5 between effects was used in these analyses.

Subsequently, the authors conducted random‐effects meta‐analyses for each adversity subtype, including for effects based on total adversity scores where available. This allowed exploration of how effects varied across adversity subtypes. Where the outcome was affective lability or emotion dysregulation, analyses used the restricted maximum‐likelihood estimator and Hartung‐Knapp adjustment^23^, ^24^ to reduce the risk of false positive results. Analyses with rapid cycling as the outcome were based on odds ratios and used the Paule‐Mandel estimator.^25^, ^26^ Analyses were undertaken in R, using the Meta package,^27^ and the Robumeta package for robust variance meta‐analysis.^22^ The *I* ^2^ statistic was used as a metric of inconsistency between studies, highlighting the proportion of variance that was between‐study. Funnel plots were produced where the number of included studies exceeded 10 to investigate the potential for publication bias.

Lastly, the review identified a small number of reports using the experience sampling method.^28^ Multilevel analysis on panel data typically has extremely small confidence intervals, which can overly influence the meta‐analytic findings, when considered alongside participant‐level data. Furthermore, there were stark differences in the variables and sampling methods employed. We therefore narratively summarised these reports, rather than including them in the meta‐analysis.

## 3 RESULTS

**FIGURE 1:**
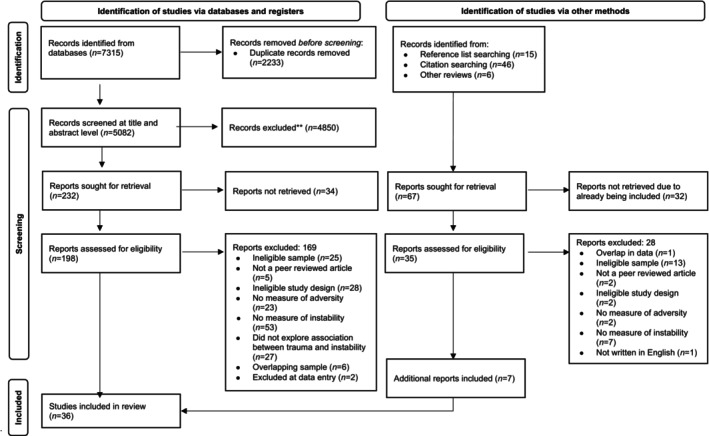
PRISMA flowchart of screening.

Figure 1 outlines the screening process. The search identified 36 reports including 8431 participants. Descriptive information is provided in Table 1. Two reports analysed data from the Fundamental Advanced Centers of Expertise in Bipolar Disorders cohort,^30^, ^57^ but focused on different outcomes (affective lability and rapid cycling) and were included but for separate analyses. Additional information was provided by 10 authors.

**TABLE 1:** Study descriptive information.

First author	Date	Sample (*n*)	Country	Age group	Status	Diagnosis system	Diagnostic measure	Instability measure	Trauma measure	Covariates
1. Affective lability										
Aas^29^	2014	BD (*n* = 42)	Norway	Adults	Outpatients and inpatients	DSM‐IV	SCID	ALS	CTQ	None
Etain^30^	2017	BD (*n* = 485)	France	Adults	Outpatients	DSM‐IV	SCID	ALS	CTQ	None
Garakani^31^	2021	BD and MDD (*n* = 109)	USA	Adults	Inpatients	DSM‐IV TR	Unclear	ALS	CTQ	None
Goodman^9^	2003	Mixed personality disorder (*n* = 174)	USA	Adults	Outpatients	DSM‐III‐R	DSM‐III‐R	ALS	CTQ	None
Marwaha^32^	2016	BDI (*n* = 923), BDII (*n* = 363), and MDD (*n* = 207)	UK	Adults	Outpatient	DSM‐IV	SCAN	ALS	CLEQ	Gender, age, education—binary, recruitment, AMS score, BDI score
Santangelo^33^	2014	PTSD (*n* = 28), BPD (*n* = 43), and BN (*n* = 20)	Germany	Adults	Outpatients and inpatients	DSM‐IV	SCID	ALS and ESM items	CTQ	None
2. Emotion dysregulation										
Bertele^34^	2022	BPD (*n* = 162)	Germany	Adults	Unclear	DSM‐IV	SCID	DERS	CTQ	None
Boger^35^	2020	OCD (*n* = 68)	Germany	Adults	Inpatients	DSM‐IV	SCID	DERS	CTQ	None
Cassioli^36^	2021	AN (*n* = 120), females	Italy	Adults	Outpatients	DSM‐V	SCID	DERS	CTQ	Model accounts for covariance with other variables
Choi^37^	2014	Mixed psychiatric (*n* = 162), not psychosis	South Korea	Adults	Outpatients	DSM‐IV	Unclear	DERS	CTQ	None
Cloitre^38^	2019	PTSD (*n* = 290), females	USA	Adults	Outpatients	DSM‐IV	CAPS‐IV/CAPS	DERS	Life stressor checklist adapted for childhood	None
Dutcher^39^	2017	Mixed psychiatric, history of trauma exposure (*n* = 111)	USA	Adults	Inpatients	DSM‐V	Life‐event checklist for DSM‐V	DERS	CTQ	None
Fernando^40^	2014	MDD (*n* = 48) and BPD (*n* = 49)	Germany	Adults	Unclear	DSM‐IV	SCID	DERS	CTQ	None
Hatkevich^41^	2021	Mixed mood, anxiety and externalising disorder (*n* = 203)	USA	Adolescents	Inpatients	DSM‐IV	CDISC	DERS	CTQ	None
Hu^42^	2023	Mixed psychiatric diagnosis (*n* = 245)	China	Adolescents	Inpatients	DSM‐V	Unclear	DERS	CTQ	None
Khosravani^43^	2021	BD (*n* = 300)	Iran	Adults	Inpatients	DSM‐V	SCID‐5‐RV	DERS	CTQ	None
Ocakoğlu^44^	2023	CDD (*n* = 3), MDD, (*n* = 67), AD (*n* = 1), adjustment disorder (*n* = 9)	Turkey	Adolescents	Outpatients	DSM‐IV	K‐SADS‐PL	DERS	CTQ	None
Peh^45^	2017	Mixed psychiatric diagnosis (*n* = 108)	Singapore	Adolescents	Outpatients	DSM‐V	Unclear	DERS	CTQ	None
Racine^46^	2015	AN (*n* = 188)	USA	Adults	Outpatients and inpatients	DSM‐IV‐TR	SCID‐I	DERS	CTQ	None
Schaefer^47^	2021	Bulimic‐spectrum disorders (*n* = 204)	USA	Adults	Outpatients	DSM‐IV	SCID‐P	DAPP‐BQ	CTQ	None
Steiger^48^	2012	BN (*n* = 196)	Canada	Adults	Outpatients	DSM‐IV‐TR	EDE	DAPP‐BQ	CTI	None
Steiger^49^	2000	BN (*n* = 35)	Canada	Adults	Outpatients	DSM‐IV	SCID‐II	DAPP‐BQ	CTI	None
Titelius^50^	2018	Mixed psychiatric diagnosis (*n* = 53)	USA	Adolescents	Inpatients	DSM‐V	KSADS‐PL	DERS	CTQ	None
Van Dijke^51^	2018	Mixed psychiatric diagnosis (*n* = 449)	Netherlands	Adults	Inpatients	DSM‐IV‐TR	SCID	BPDSI and SIDES‐rev‐NL	TEC Dutch Version	None
Wang^52^	2021	Depression (*n* = 496)	China	Adults	Inpatients	ICD‐10	Unclear	ERS	ACE‐IQ	None
Yang^53^	2021	BDI (*n* = 56), BDII (*n* = 104), and MDD (*n* = 31)	South Korea	Adults	Outpatients	DSM‐V	Unclear	DERS	CTQ	None
Zlotnick^54^	2001	MDD (*n* = 235)	USA	Adults	Outpatients	DSM‐IV	SCID‐I	SIDES	SCID module for PTSD	BPD and PTSD status
3. Rapid cycling										
Brown^55^	2005	Veterans with BD (*n* = 180)	USA	Adults	Outpatients	DSM‐IV	SCID	Clinical interview	Own interview schedule	None
de Azambuja Farias^56^	2019	BD (*n* = 90)	Brazil	Young adults	Outpatients	DSM‐IV	MINI‐PLUS and SCID	MINI‐PLUS	CTQ	None
Etain^57^	2013	BDI (*n* = 425), BDII (*n* = 126), and BD NOS (*n* = 36)	Norway and France	Adults	Outpatients and inpatients	DSM‐IV	SCID‐I	DIGS	CTQ	None
Garno^58^	2005	BD (*n* = 99)	USA	Adults	Outpatients and inpatients	DSM‐IV	SCID‐IV	SCID‐IV	CTQ	None
Jaworska‐Andryszewska^59^	2018	BD‐I (*n* = 41) and BD‐II (*n* = 11)	Poland	Adults	Outpatients and inpatients	ICD‐10	Consensus by two psychiatrists	Clinical interview	CTQ	None
McIntyre^60^	2008	BD (*n* = 381)	Canada	Adults	Outpatients	DSM‐IV‐TR	Clinical interview	Clinical interview	Clinical interview	None
Perich^61^	2014	BD (*n* = 157)	Australia	Adults	Outpatients	DSM‐IV‐TR	SCID‐I	SCID‐I	Own interview schedule	None
Post^62^	2013	BD (*n* = 850)	USA, Netherlands, and Germany	Adults	Outpatients	DSM‐IV	SCID‐P	Clinician Questionnaire	Clinician Questionnaire	None
4. Experience sampling										
Brick^63^	2021	Mixed psychiatric diagnosis (*n* = 133)	USA	Adults	Inpatients	DSM	SCID	ESM items	CTQ	Time, whether report was self‐initiated

All studies were published on or after the year 2000, with the majority published in the last 10 years at the time of writing (75%; *k* = 27). One study included a 1‐year follow‐up^36^ but we were only able to extract effect size information from the baseline data. All extracted effect size information was therefore cross‐sectional. We did not identify any prospective longitudinal research. Most studies included adult samples (83%; *k* = 30) from Europe and the United States of America (68%; *k* = 24). Almost half of the studies focussed on people with bipolar disorder or borderline personality disorder (49%; *k* = 17), 51% (*k* = 18) recruited outpatients and 27% (*k* = 10) recruited inpatients; the remaining studies either recruited both outpatients and inpatients or were unclear. All reports utilised the DSM or ICD diagnostic system, as per our inclusion criteria, and most used a variation of the SCID as the diagnostic measure (55%; *k* = 20). 70% (*k* = 25) of the studies utilised the Childhood Trauma Questionnaire^64^ to measure adversity.

### 3.1 Affective lability

The correlated‐effects model suggested a significant, but small, effect of adversity on affective lability (*r* = 0.09; 95% CI = 0.02, 0.17; 10 samples, 44 effects; *I* ^2^ = 61.09%). A forest plot of these results is displayed in Supplementary Figure 1. Effects varied considerably by adversity subtype. The analysis was repeated excluding three samples from one study where only adjusted associations were available,^32^ leading to a slightly larger, but still small, pooled effect (*r* = 0.15; 95% CI = 0.05, 0.25; 7 samples, 32 effects; *I* ^2^ = 45.53%). A funnel plot did not suggest any publication bias.

### 3.2 Emotional dysregulation

The correlated‐effects model suggested a significant, but small, effect of adversity on emotion dysregulation (*r* = 0.25, 95% CI = 0.19; 0.32; 22 samples, 57 effects; *I* ^2^ = 80.21%), which is displayed in Supplementary Figure 2. As with affective lability, effects varied considerably by adversity type. The analysis was repeated excluding two effect sizes from two studies^36^, ^54^ where only adjusted associations were available, which made minimal difference to the results (*r* = 0.27; 95% CI = 0.20, 0.34; 20 studies, 55 effects; *I* ^2^ = 80.55%). A funnel plot suggested slight asymmetry in the plot running in the counter direction to what would indicate bias, with studies characterised by less variance encompassing larger effects.

### 3.3 Rapid cycling

As shown in Supplementary Figure 3, the correlated‐effects model suggested a significant and small effect of adversity on rapid cycling (OR = 1.39; 95% CI = 1.14, 1.70; 8 samples, 18 effects; *I* ^2^ = 41.57%). For one study^59^ the reported confidence intervals, when converted to the log scale, were not symmetrical as would be expected so the analysis was repeated with this one effect removed, which made minimal difference to the findings (OR = 1.40; 95% CI = 1.15, 1.71; 8 samples, 17 effects; *I* ^2^ = 33.38%). A funnel plot did not provide any evidence of publication bias.

### 3.4 Adversity subtype analysis

Separate random‐effects meta‐analyses were undertaken for each adversity subtype for each outcome, with the results presented in Table 2. Emotional abuse had the largest effects on affective lability (*r* = 0.16, 95% CI 0.07–0.24) and emotional dysregulation (*r* = 0.32, 95% CI 0.19–0.44). Physical abuse had the largest effects on rapid cycling (OR: 2.49, 95% CI 1.30–4.78), but the small number of studies means that this finding should be treated with caution. There was high inconsistency for most analyses suggesting that point estimates of pooled effects should be treated with caution as important between‐study differences may exist. A sensitivity analysis was completed where reports with adjusted effects were removed, which yielded slightly larger effects, particularly when considering affective lability as an outcome.

**TABLE 2:** Adversity subtype analysis.

Outcome	Adversity type	Adjusted effects included				Adjusted effects removed			
		*k*	*r*	CI	*I* ^2^ (%)	*k*	*r*	CI	*I* ^2^ (%)
Affective lability	Emotional abuse	10	0.16	0.07, 0.24	63.8	7	0.25	0.19 0.31	0
	Physical abuse	10	0.08	−0.08, 0.16	47	7	0.12	−0.02, 0.25	41.4
	Sexual abuse	9	0.08	0.01, 0.14	21.8	6	0.14	0.05, 0.23	0
	Emotional neglect	6	0.13	−0.12, 0.36	62.9	‐	‐	‐	
	Physical neglect	6	0.1	−0.05, 0.24	12.9	‐	‐	‐	
	Total/any adversity	7	0.07	−0.01, 0.16	43.1	4	0.31	0.11, 0.48	0

		*k*	*r*	CI	*I* ^2^ (%)	*k*	*r*	CI	*I* ^2^ (%)
Emotion dysregulation	Emotional abuse	10	0.32	0.19, 0.44	84.7	‐	‐	‐	‐
	Physical abuse	10	0.15	0.02, 0.27	78.2	‐	‐	‐	‐
	Sexual abuse	12	0.11	0.07, 0.15	0	11	0.12	0.07, 0.16	0
	Emotional neglect	8	0.26	0.09, 0.42	86.5	‐	‐	‐	
	Physical neglect	7	0.17	0.02, 0.31	77.2	‐	‐	‐	
	Total/any adversity	12	0.27	0.17, 0.36	68.3	11	0.27	0.17, 0.37	70.7

		*k*	OR	CI	*I* ^2^ (%)	*k*	OR	CI	*I* ^2^ (%)
Rapid cycling	Emotional abuse	3	2.12	0.95, 4.85	83.7	‐	‐	‐	‐
	Physical abuse	2	2.49	1.30, 4.78	27	‐	‐	‐	‐
	Sexual abuse	3	1.57	0.95, 2.58	57.5	‐	‐	‐	‐
	Emotional neglect	3	1.71	0.92, 3.18	69.4	2	2.25	0.94, 5.36	62.0
	Physical neglect	2	0.68	0.37, 1.27	0	‐	‐	‐	‐
	Total/any adversity	6	1.25	1.05, 1.55	51.3	‐	‐	‐	‐

One study explored the association between the death of a parent and affective lability in participants with Bipolar I, Bipolar II and Major Depressive Disorder with small correlations ranging from *r* = −0.04 to =0.06, when controlling for a range of covariates. We did not find any studies focusing on childhood bullying.

### 3.5 Experience sampling method

Two reports explored the association between childhood adversity and affective instability using the experience sampling method, which were not meta‐analysed. There were distinct methodological differences between these studies. Brick and colleagues^63^ asked 133 participants to complete five assessments of affect per day for a 3‐week sampling period. Emotional, physical, and sexual abuse were not associated with within‐person variance in either positive or negative affect, with small β ranging from −0.01 to 0.03. Santengelo and colleagues^33^ asked participants to rate a range of emotional states every 15 min for 24 h and calculated the squared successive difference (SSD), which was not significantly associated with total trauma in patients with post‐traumatic stress disorder (*r* = 0.13, *p* = 0.518, *n* = 26), borderline personality disorder (*r* = −0.07, *p* = 0.677, *n* = 41), or bulimia nervosa (*r* = 0.30, *p* = 0.234, *n* = 18), although the sample sizes were small. Of the adversity subtypes, only childhood neglect was associated with the SSD score, and only in the post‐traumatic stress disorder (*r* = 0.39, *p* = 0.048, *n* = 26) and borderline personality disorder (*r* = 0.30, *p* = 0.047, *n* = 43) samples.

### 3.6 Quality assessment

The results to quality assessment are presented in Supplementary Table 2. To summarise, quality assessment ratings (out of 5) were generally low to moderate (mean 2.8, SD 0.6, range 1–4). Only three studies (9%) were deemed to have evidence of a representative sample. Only two studies (6%) controlled for socioeconomic status, age, and gender. However, 80% of studies had used validated outcome measures and 83% had complete outcome data.

## 4 DISCUSSION

This systematic review was the first to meta‐analyse the association between childhood adversity and emotional instability. The analyses suggest a small, but statistically significant, association between childhood adversity and affective instability. This should be interpreted in the context of no prospective longitudinal or dose response studies, and low‐moderate quality assessment scores. Nevertheless, this finding was relatively robust and observed across all three affective instability outcomes.

Of the adversity subtypes, emotional abuse had the strongest association with affective lability and emotion dysregulation. This is consistent with research proposing links between emotional invalidation and rejection sensitivity,^65^ and past meta‐analyses showing strong associations between childhood emotional abuse and the diagnosis of disorders characterised by affective lability.^16^, ^17^ Emotional adversity may play an important role in shaping the regulation and volatility of emotions later in life. This could be explained by emotional abuse being particularly likely to result in the internalisation of negative messages from others (e.g., self as worthless), which may leave some individuals more vulnerable to dysregulated emotions. At times, emotional abuse can occur in the context of close attachment relationships,^66^ which may amplify its negative effects. It may also be a co‐occurring component of many other forms of abuse (e.g., physical or sexual) and may exacerbate the impact of these experiences. Further largescale, prospective design research is needed to understand whether there is a causal link between emotional adversity and affective instability.

### 4.1 Limitations

This meta‐analysis was restricted to reports in the English language and peer reviewed journals, but did involve a comprehensive search of the available literature. The number of analysable reports was relatively small, particularly when considering the impact of adversity subtypes on rapid cycling. Most studies did not control for key covariates (e.g., age, gender, current or historical socioeconomic status), which may have inflated the size of the effects. However, removing studies controlling for covariates from the analysis did not greatly change the results of the current meta‐analysis.

Experience sampling research^28^ may be particularly suited to the study of affective instability. The two identified studies^33^, ^63^ found non‐significant and small effects which were not meta‐analysed. One of these studies had a modest sample size.^33^ This represents a clear and important area for exploration in future research. All identified studies employed a retrospective measure of childhood adversity, which may be subject to recall bias. The search failed to identify any papers exploring the impact of childhood bullying on affective instability outcomes. Further work is needed to understand the association between affective instability and other forms of childhood adversity. It may also be important to understand the timing of adversity and whether abuse was inter‐ or intra‐familial, to determine their impact on affective instability outcomes.

The authors note two deviations from the original review protocol. First, we had planned to use the Newcastle Ottawa Assessment Scale as the quality assessment. However, it is better suited for case control studies, none of which were identified in the review. The authors therefore employed the Mixed‐Methods Appraisals Tool instead. Second, calculating a global, overall effect size (one effect per report) can be problematic and less meaningful when the strength of the effect varies considerably across variables, which was true for the adversity subtypes in this review. Therefore, rather than calculating a global effect, we used a random correlated‐effects model with small sample size correction, which uses robust variance estimation, to allow for non‐independent effects to be included together. This was seen as a more robust and valid approach to analysing this data.

### 4.2 Clinical implications

The findings support a move towards trauma‐informed care in psychiatric patients experiencing high levels of affective instability, with particular emphasis on treating the adverse effects of emotional abuse. For example, within cognitive behavioural therapy, this might involve supporting people to understand how their past experiences shape their current emotional states and regulation strategies, to recalibrate potentially unhelpful coping responses.^67^ Emotional abuse in childhood may represent a sustained, extreme, and negative pattern of interaction. Children experiencing emotional abuse may experience low self‐esteem, difficulties making friends, and depression.^68^ Better early identification and intervention of emotional abuse may represent a putative mechanism for preventing difficulties with affective instability later in life. Emotional abuse may be more challenging to identify than more overt forms of abuse, often escaping the notice of clinical and social care services, and this may make it more insidious and harder to prevent. Past research has suggested that high affective instability may be associated with adverse clinical outcomes, including functioning^3^ and suicidal ideation.^69^ Campaigns that support the recognition and prevention of emotional abuse may therefore be important.

To conclude, this meta‐analysis observed a small, but statistically significant, effect of childhood adversity on different metrics of affective instability later in life. Childhood emotional abuse showed the strongest association with affective lability and emotion dysregulation, highlighting the importance of trauma informed care. Further prospective longitudinal research is needed to explore whether a causal relationship exists.

## CONFLICT OF INTEREST STATEMENT

The authors can confirm that there are no competing interests arising from this research. Template data extraction forms are available from the research team upon request.

## Supporting information


**Data S1** Supporting Information.

## Data Availability

The data that support the findings of this study are available from the corresponding author upon reasonable request.
