# Supplementary material for: The relationship between childhood adversity and affective instability across psychiatric disorders: A meta‐analysis
Source: Acta Psychiatr Scand. 2024 Aug 11;151(1):33–45. doi: 10.1111/acps.13745 (PMC11608813; doi:10.1111/acps.13745)
Supplement: Supplementary file 1 — Data S1 Supporting Information. [file ACPS-151-33-s001.docx]

Supplementary Table 1. Search terms.

| Search engine | Search terms |
| --- | --- |
| **Medline** | 1. (MH "Child Abuse+") OR (MH "Adult Survivors of Child Abuse") OR (MH "Child Abuse, Sexual") OR (MH "Child") OR (MH "Child, Preschool") OR "child* abuse" OR "child* maltreatment" OR “child* trauma” 2. ((MH "Physical Abuse") OR (MH "Sex Offenses") OR (MH "Bullying") Or (MH "Crime Victims") OR (MH "Parental Death") OR “physical abuse” or “sexual abuse” or “emotional abuse” or neglect* or trauma* or advers* or maltreat* or bully* or bullied or victim* or “parental loss”)) 3. affect* or emotion* or mood 4. instability or unstable or labil* or regulat* or dysregulat* or fluctuat* or cycling 5. S1 AND S2 AND S3 AND S4 6. Limit to ‘English’ 7. Limit to ‘1980 – 2020’ 8. Limit to ‘Human’ |
| **PsychInfo** | 1. DE "Child Abuse" OR MM "Child Abuse Reporting" OR MM "Child Neglect" OR MM "Child Welfare" OR "child* abuse" OR "child* maltreatment" OR “child* trauma” 2. MM "Emotional Abuse" OR MM "Verbal Abuse" OR MM "Sexual Abuse" OR MM "Physical Abuse" OR MM "Parental Death" OR “physical abuse” or “sexual abuse” or “emotional abuse” or neglect* or trauma* or advers* or maltreat* or bully* or bullied or victim* or “parental loss” 3. Affect* or emotion* or mood 4. instability or unstable or labil* or regulat* or dysregulat* or fluctuat* or cycling 5. S1 AND S2 AND S3 AND S4 6. Limit to ‘English’ 7. Limit to ‘1980 – 2020’ 8. Limit to ‘Human’ |
| **Embase** | 1. exp childhood adversity/ or ‘child$ abuse’.mp. or ‘child$ maltreatment’.mp. or ‘child$ trauma’.mp. 2. ('physical abuse' or 'sexual abuse' or 'emotional abuse' or neglect$ or trauma$ or advers$ or maltreat$ or bully$ or bullied or victim$ or 'parental loss').mp. 3. (affect$ or emotion$ or mood).mp. 4. (instability or unstable or labil$ or regulat$ or dysregulat$ or fluctuat$ or cycling).mp. 5. 1 AND 2 AND 3 AND 4 6. limit 5 to English language 7. limit 6 to yr="1980 -Current" 8. limit 7 to human |

Supplementary Figure 1. A forest plot showing the effect of childhood adversity on affective lability.


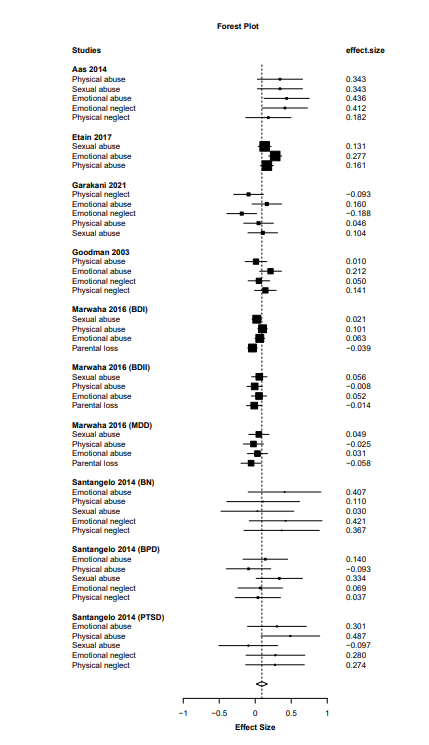


Figure 1 Key: BDI - Bipolar Disorder I, BDII - Bipolar Disorder II, BPD - Borderline Personality Disorder, BN - Bulima Nervosa, MDD - Major Depressive Disorder, PTSD - Post-Traumatic Stress Disorder.

Supplementary Figure 2. A forest plot of the effect of childhood adversity on emotional dysregulation.


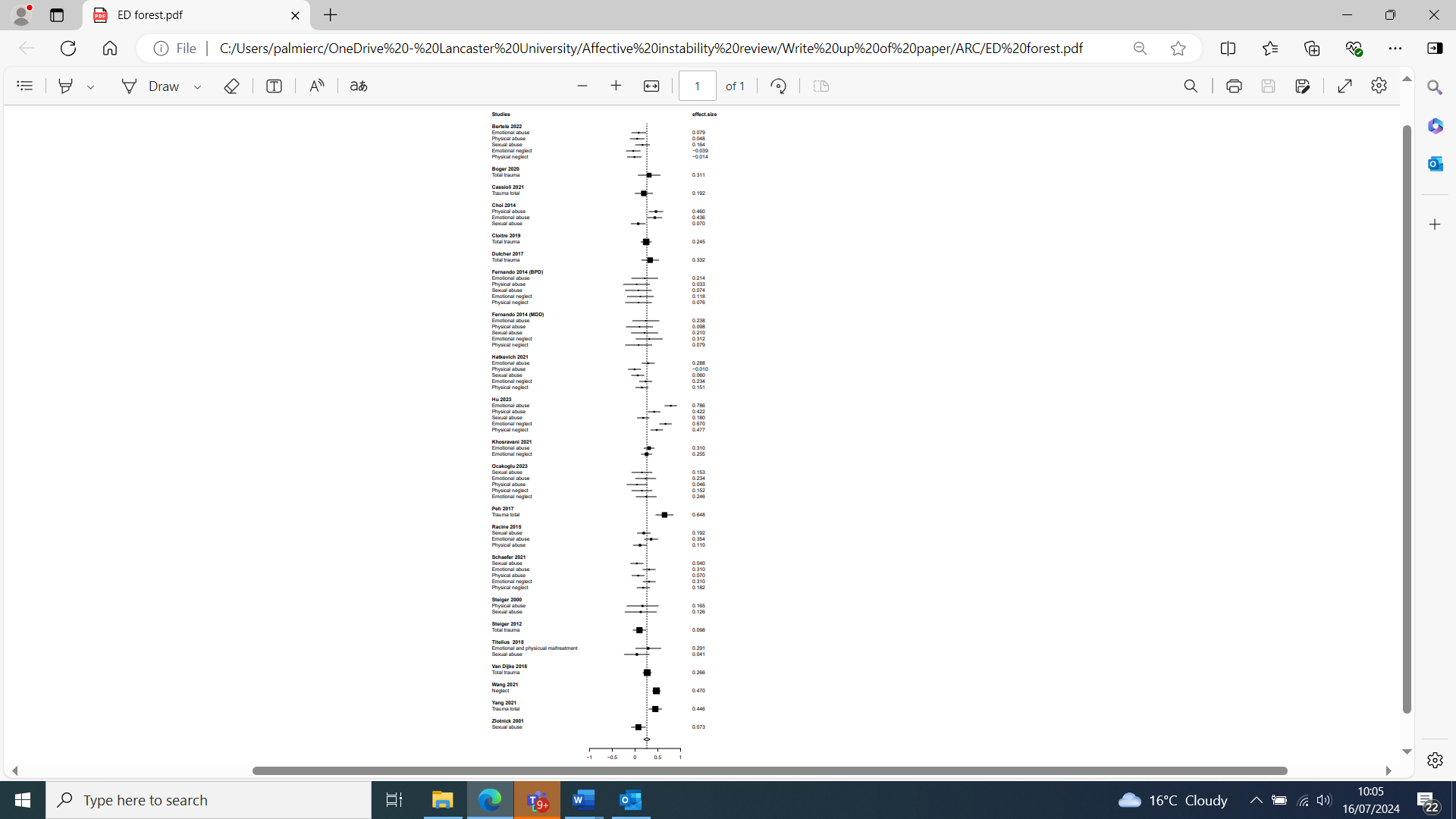


Figure 2 Key: BPD – Borderline Personality Disorder, MDD – Major Depressive Disorder

Supplementary Figure 3. A forest plot showing the effect of childhood adversity on rapid cycling.


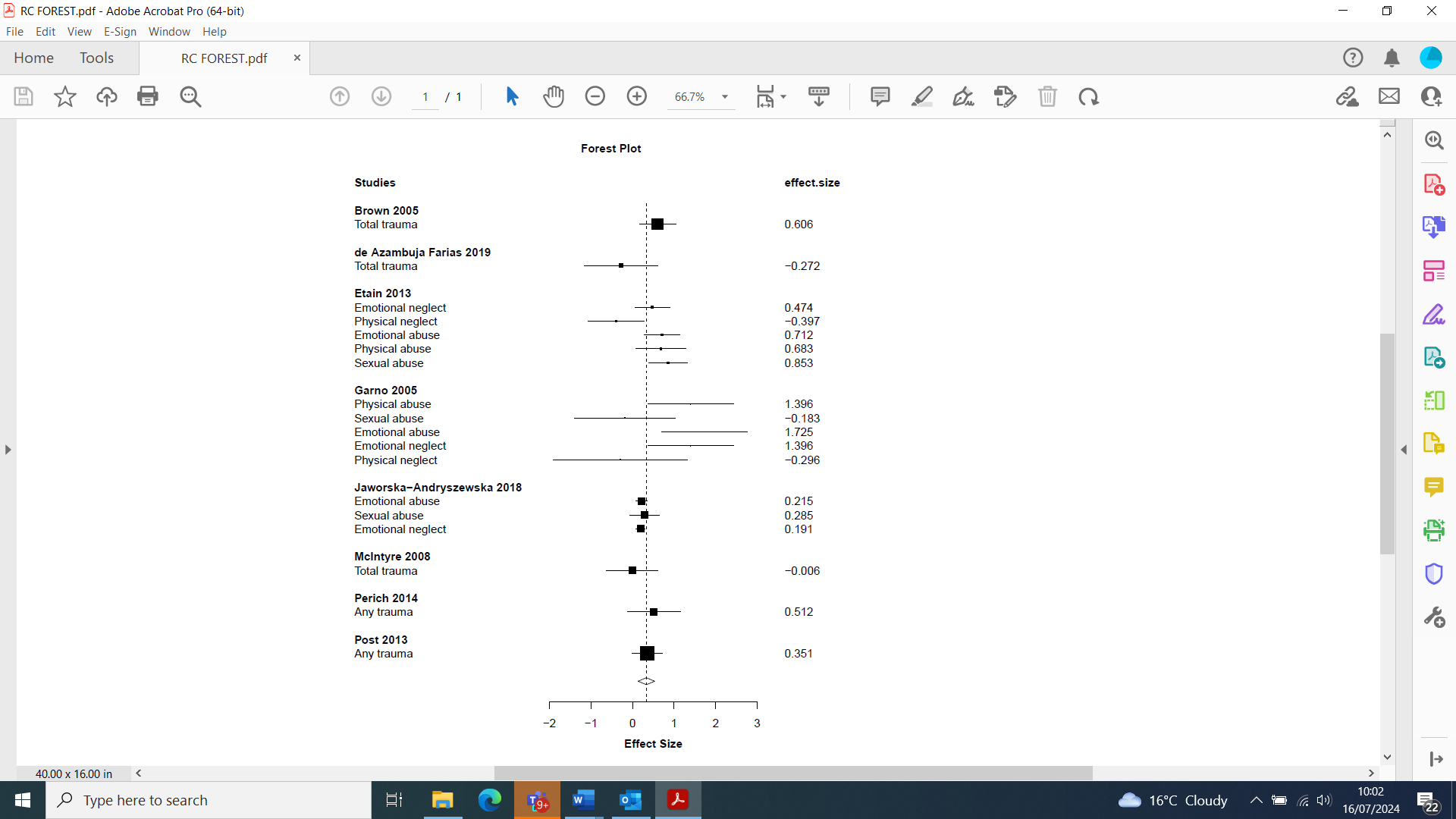


Supplementary Table 2. Quality assessment using the Mixed Methods Appraisal Tool (MMAT).

|  | 1. Representativeness of sample | 2. Appropriate measure of exposure | 3. Complete outcome data | 4. Accounting for confounders | 5. Exposure occurred as intended? | Total score |
| --- | --- | --- | --- | --- | --- | --- |
| Aas (2014) | 0 | 1 | 1 | 0 | 1 | 3 |
| Bertele (2022) | 0 | 1 | 1 | 0 | 1 | 3 |
| Boger (2020) | 0 | 1 | 1 | 0 | 1 | 3 |
| Brick (2021) | 0 | 1 | 0 | 0 | 1 | 2 |
| Brown (2005) | 0 | 0 | 1 | 0 | 1 | 2 |
| Cassioli (2021) | 0 | 1 | 1 | 0 | 1 | 3 |
| Choi (2015) | 0 | 1 | 1 | 0 | 1 | 3 |
| Cloitre (2019) | 0 | 0 | 1 | 0 | 1 | 2 |
| de Azambuja Farias (2019) | 1 | 1 | 0 | 1 | 1 | 4 |
| Dutcher (2017) | 0 | 1 | 1 | 0 | 1 | 3 |
| Etain (2013); also Etain (2017) | 0 | 1 | 1 | 0 | 1 | 3 |
| Fernando (2014) | 0 | 1 | 1 | 0 | 1 | 3 |
| Garakani (2021) | 0 | 1 | 1 | 0 | 1 | 3 |
| Garno (2005) | 0 | 1 | 1 | 0 | 1 | 3 |
| Goodman (2003) | 0 | 1 | 1 | 0 | 1 | 3 |
| Hatkevich (2021) | 1 | 1 | 0 | 0 | 1 | 3 |
| Hu (2023) | 0 | 1 | 1 | 0 | 1 | 3 |
| Jakworska-Andryszewska (2018) | 0 | 0 | 1 | 0 | 1 | 2 |
| Khosravani (2021) | 0 | 1 | 1 | 0 | 1 | 3 |
| Marwaha (2016) | 0 | 0 | 1 | 1 | 1 | 3 |
| McIntyre (2008) | 0 | 0 | 0 | 0 | 1 | 1 |
| Ocakoğlu (2023) | 0 | 1 | 1 | 0 | 1 | 3 |
| Peh (2017) | 0 | 1 | 1 | 0 | 1 | 3 |
| Perich (2014) | 0 | 0 | 0 | 0 | 1 | 1 |
| Post (2013) | 0 | 0 | 1 | 0 | 1 | 2 |
| Racine & Wildes (2015) | 0 | 1 | 1 | 0 | 1 | 3 |
| Santangelo (2014) | 0 | 1 | 1 | 0 | 1 | 3 |
| Schaefer (2021) | 0 | 1 | 1 | 0 | 1 | 3 |
| Steiger (2000) | 0 | 1 | 1 | 0 | 1 | 3 |
| Steiger (2012) | 0 | 1 | 1 | 0 | 1 | 3 |
| Titelius (2018) | 0 | 1 | 1 | 0 | 1 | 3 |
| Van Dijke (2018) | 0 | 1 | 1 | 0 | 1 | 3 |
| Wang (2021) | 1 | 1 | 1 | 0 | 1 | 4 |
| Yang (2021) | 0 | 1 | 1 | 0 | 1 | 3 |
| Zlotnick (2001) | 0 | 1 | 0 | 0 | 1 | 2 |
